# Early Embryogenesis-Specific Expression of the Rice Transposon *Ping* Enhances Amplification of the MITE *mPing*

**DOI:** 10.1371/journal.pgen.1004396

**Published:** 2014-06-12

**Authors:** Shota Teramoto, Takuji Tsukiyama, Yutaka Okumoto, Takatoshi Tanisaka

**Affiliations:** 1 Division of Agronomy and Horticulture Science, Graduate School of Agriculture, Kyoto University, Sakyo, Kyoto, Japan; 2 Department of Agriculture for Regional Reclamation, Kibi International University, Minami-Awaji, Japan; University of Utah School of Medicine, United States of America

## Abstract

Miniature inverted-repeat transposable elements (MITEs) are numerically predominant transposable elements in the rice genome, and their activities have influenced the evolution of genes. Very little is known about how MITEs can rapidly amplify to thousands in the genome. The rice MITE *mPing* is quiescent in most cultivars under natural growth conditions, although it is activated by various stresses, such as tissue culture, gamma-ray irradiation, and high hydrostatic pressure. Exceptionally in the temperate *japonica* rice strain EG4 (cultivar Gimbozu), *mPing* has reached over 1000 copies in the genome, and is amplifying owing to its active transposition even under natural growth conditions. Being the only active MITE, *mPing* in EG4 is an appropriate material to study how MITEs amplify in the genome. Here, we provide important findings regarding the transposition and amplification of *mPing* in EG4. Transposon display of *mPing* using various tissues of a single EG4 plant revealed that most *de novo mPing* insertions arise in embryogenesis during the period from 3 to 5 days after pollination (DAP), and a large majority of these insertions are transmissible to the next generation. Locus-specific PCR showed that *mPing* excisions and insertions arose at the same time (3 to 5 DAP). Moreover, expression analysis and *in situ* hybridization analysis revealed that *Ping*, an autonomous partner for *mPing*, was markedly up-regulated in the 3 DAP embryo of EG4, whereas such up-regulation of *Ping* was not observed in the *mPing*-inactive cultivar Nipponbare. These results demonstrate that the early embryogenesis-specific expression of *Ping* is responsible for the successful amplification of *mPing* in EG4. This study helps not only to elucidate the whole mechanism of *mPing* amplification but also to further understand the contribution of MITEs to genome evolution.

## Author Summary

Transposable elements are major components of eukaryotic genomes, comprising a large portion of the genome in some species. Miniature inverted-repeat transposable elements (MITEs), which belong to the class II DNA transposable elements, are abundant in gene-rich regions, and their copy numbers are very high; therefore, they have been considered to contribute to genome evolution. Because MITEs are short and have no coding capacity, they cannot transpose their positions without the aid of transposase, provided *in trans* by their autonomous element(s). It has been unknown how MITEs amplify themselves to high copy numbers in the genome. Our results demonstrate that the rice active MITE *mPing* is mobilized in the embryo by the developmental stage-specific up-regulation of an autonomous element, *Ping*, and thereby successfully amplifies itself to a high copy number in the genome. The short-term expression of *Ping* is thought to be a strategy of the *mPing* family for amplifying *mPing* by escaping the silencing mechanism of the host genome.

## Introduction

Transposable elements (TEs) are DNA sequences that are capable of jumping from one genomic locus to another and make up a large fraction of eukaryotic genomes. More than 80% of the maize (*Zea mays*) and barley (*Hordeum vulgare*) genomes are composed of TEs [1], [2], and they constitute 35% and 14% of the genomes of rice (*Oryza sativa*) and Arabidopsis (*Arabidopsis thaliana*), respectively [3], [4]. TEs are harmful to the host because their mobilities perturb genome stability, whereas they play greatly generative roles in genome evolution such as alternation of gene structure, change of expression pattern, and rearrangement of chromosome structure [5], [6].

TEs are classified into two groups according to their transposition mechanisms: class I elements (retrotransposons) that transpose through a copy-and-paste mechanism via an RNA intermediate, and class II elements (transposons) that transpose through a cut-and-paste mechanism without undergoing an RNA intermediate. Class I elements easily attain tens of thousands of copies, whereas the majority of class II elements cannot amplify themselves to 50 copies at most. Unlike other class II elements, miniature inverted-repeat transposable elements (MITEs) have the capacity to amplify themselves to high copy numbers (hundreds or thousands) [7]–[9]. In the rice genome, MITEs are numerically predominant TEs [10], constituting 8.6% of the genome [11]. Because MITEs are too short (<600 bp) to encode any protein, their transpositions must depend on the proteins encoded by the autonomous elements. Well-studied MITEs are classified into the *Stowaway* and *Tourist* families, which belong to the *Tc1*/*mariner* and *PIF*/*Harbinger* superfamilies, respectively. Because MITEs are mainly deployed in gene-rich regions [10], [12] and affect adjacent gene expression [13], they are considered to play an important role in genome evolution. However, little is known about how MITEs attain high copy numbers.

*Miniature Ping* (*mPing*) is the first active MITE identified in the rice genome [14]–[16]. Although MITEs are deployed in the genome at a high copy number, the copy number of *mPing* exceptionally remains at a low level in most rice cultivars: *indica* and tropical *japonica* cultivars have fewer than 10 copies, and temperate *japonica* cultivars including Nipponbare have approximately 50 copies [14]. The transposition of *mPing* is suppressed in most rice cultivars, but, like other TEs, *mPing* is activated by exposure to various stress conditions such as gamma-ray irradiation [16], hydrostatic pressurization [17], cell culture [14], anther culture [15], and inhibition of topoisomerase II [18]. Introgression of distantly related genomes also causes *mPing* transposition [19], [20]. However, *mPing* is actively transposing without such stresses in the temperate *japonica* rice strain EG4 (cultivar Gimbozu) under natural growth conditions, and its copy number is approximately 1000 copies [21]. This indicates that *mPing* has overcome the silencing mechanism or established a novel strategy for its amplification in the EG4 genome. In this sense, *mPing* in EG4 is an appropriate material to study the amplification of MITEs in plant genomes.

The autonomous element *Ping* and its distantly related element *Pong*, which both belong to the *PIF*/*Harbinger* superfamily, provide two proteins required for *mPing* transposition. Both *Ping* and *Pong* have two open reading frames (ORFs), ORF1 and ORF2 [22], [23]. The former encodes a Myb-like DNA-binding protein, and the latter encodes a transposase lacking DNA binding domain. Transposase of most class II elements contains a conserved catalytic domain (DDE motif) and a DNA-binding domain [23], [24], whereas these domains are encoded separately by two ORFs in both *Ping* and *Pong* [22], [23]. The study of other members of the *PIF*/*Harbinger* superfamily suggested that the Myb-like DNA-binding protein directly binds to the subterminal regions of the transposon in order to recruit the transposase [25]. Both Myb-like protein and transposase of either *Ping* or *Pong* or both elements are necessary for *mPing* transposition [22], [23].

In this study, we demonstrate that *mPing* is actively transposing in the embryo of EG4 during the period from the regionalization of shoot apical meristem (SAM) and radicle to the formation of the first leaf primordium (3 to 5 days after pollination, DAP) with the aid of developmental stage-specific expression of *Ping*. Our results provide important evidence for the amplification mechanism not only of *mPing* but also of other MITEs.

## Results

### Transpositions of *mPing* during gametogenesis

**Figure 1:**
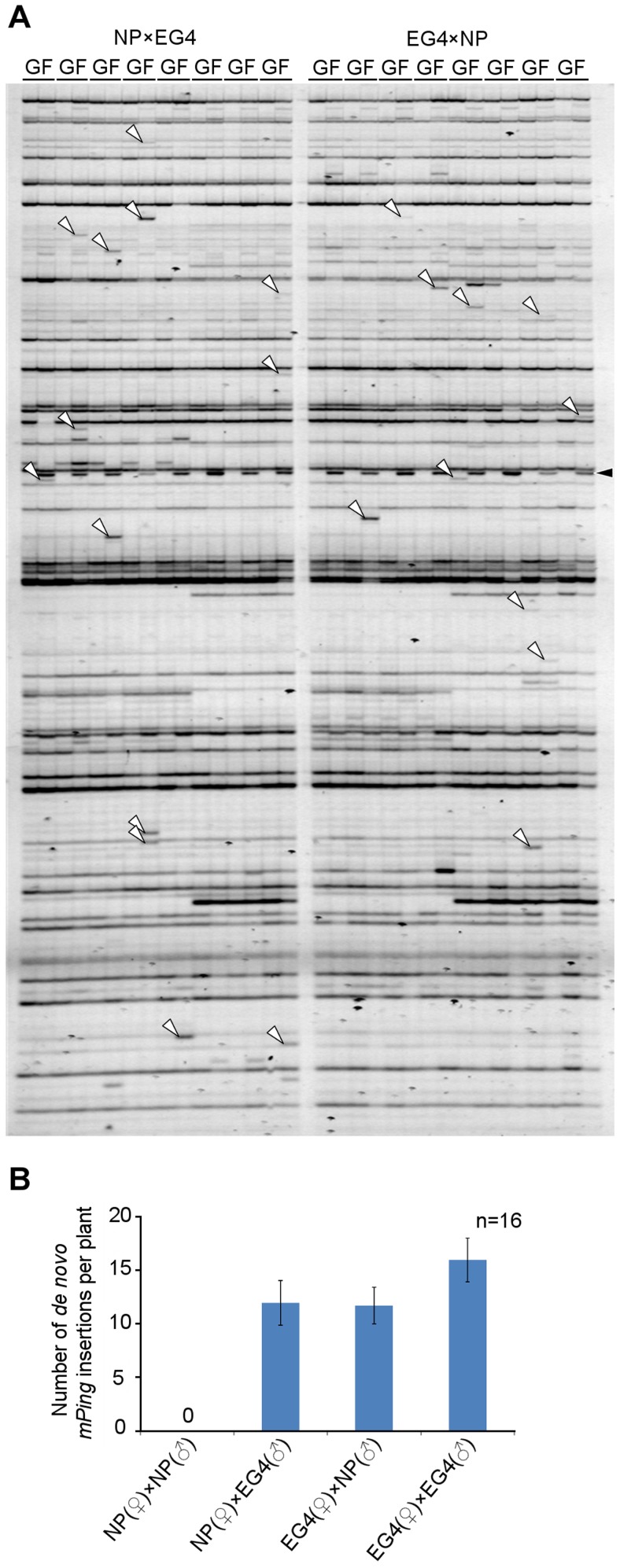
Transposition of *mPing* in reciprocal crosses between EG4 and Nipponbare. (A) Transposon display (TD) for *mPing* of the F_1_ population from reciprocal crosses between EG4 and Nipponbare. One of the results of TD analysis using two selective bases is shown. The cross combinations are indicated at the top of the profiles, respectively. G and F indicate parental EG4 and the F_1_ plants, respectively. White and black arrowheads indicate the bands representing the *de novo mPing* insertion and the band derived from Nipponbare genome, respectively. (B) Mean numbers of *de novo mPing* insertions in a single F_1_ plant and in a self-pollinated plant. The cross combinations are indicated at the bottom of the profile. All 16 possible primer combinations were analyzed, and mean values were calculated using 16 individuals (n = 16). Bars indicate SE.

Plants have acquired the silencing mechanism of TEs in germ cells. In Arabidopsis, for example, TEs are activated specifically in the vegetative nucleus of the pollen, and siRNAs from the activated TEs accumulate in the sperm cells [26]. On the basis of these results, Slotkin and colleagues proposed that siRNAs derived from TEs activated in the vegetative nucleus silence TEs in the sperm cells [26]. We conceived that *mPing* might overcome such a silencing mechanism in EG4. To confirm this hypothesis, we developed two F_1_ populations from reciprocal crosses between the *mPing*-active strain EG4 and the *mPing*-inactive cultivar Nipponbare, and investigated the transposition activity of *mPing* by transposon display (TD) analysis. Success of reciprocal crosses was confirmed by PCR analysis using locus-specific primers (Figure S1A). One of the results of TD analysis using two selective bases is shown in Figure 1A; all 16 possible primer combinations were analyzed. The banding patterns of F_1_ plants were almost the same as those of EG4. The bands that appeared in all F_1_ plants but not in the parental EG4 plant were derived from another parental Nipponbare plant (Figure S1B). Furthermore, the bands that appeared in only one of eight F_1_ plants but not in the parental EG4 plant are herein referred as *de novo* insertions. These bands were confirmed not to be PCR artifacts by sequence and locus-specific PCR analysis (Table S1 and Figure S2). We detected 15.5 *de novo* insertions per plant in the selfed progenies of EG4, whereas Nipponbare yielded no *de novo* insertions in the selfed progenies (Figure 1B). This confirmed that *mPing* is active in EG4 under natural growth conditions but inactive in Nipponbare. If *mPing* was specifically activated in the pollen of EG4, we could obtain *de novo* insertions only in the F_1_ plants from the Nipponbare/EG4 cross. However, we obtained *de novo* insertions in both Nipponbare/EG4 and EG4/Nipponbare populations (Figure 1B). Moreover, there was no significant difference in the number of *de novo* insertions per plant between the two F_1_ populations. This indicates that the activating factor(s) for the *mPing* transposition is present in both male and female gametes of EG4.

### Transpositions of *mPing* during ontogeny of EG4 plants

**Figure 2:**
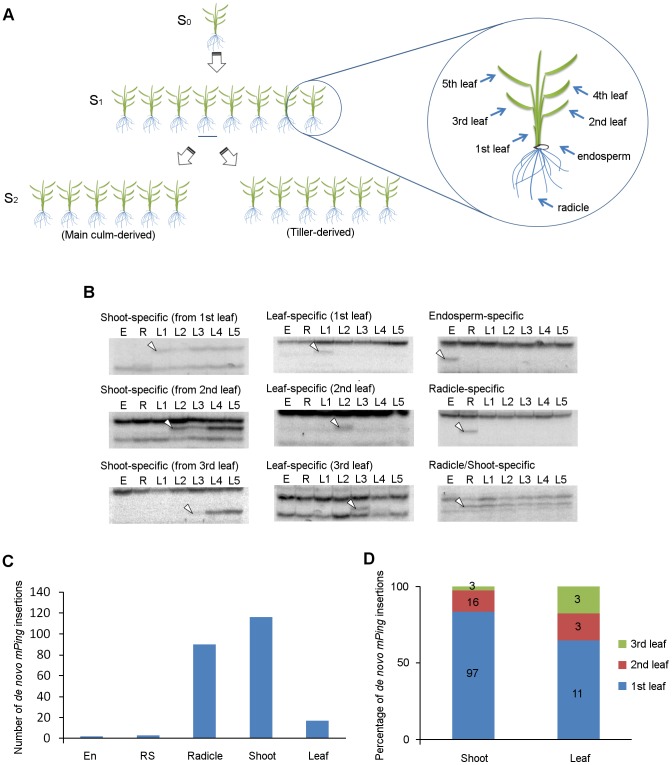
*De novo mPing* insertions during rice ontogeny. (A) Experimental setup for the ontogenical analysis to determine the timing of *mPing* transposition in EG4. Eight progenies (S_1_) derived from a single parental EG4 plant (S_0_) were grown in a greenhouse. Endosperm, radicle, and leaf blades (1st to 5th) of each S_1_ plant were sampled for DNA extraction. S_2_ seeds were harvested from the main culm and the primary tiller of each S_1_ plant to investigate the inheritance of *de novo mPing* insertions. The 2nd leaf blade of S_0_ and S_2_ plants was also sampled. Detailed information is provided in the ‘Materials and Methods’. (B) *mPing* insertions were detected by transposon display. Representative images of shoot-, leaf-, endosperm-, radicle-, and radicle/shoot-specific insertions are shown. White arrowheads indicate the bands representing the *de novo mPing* insertion. E: endosperm, R: radicle, L1–L5: 1st to 5th leaf blades. (C) The number of organ-specific *de novo* insertions in EG4. All 16 possible primer combinations were analyzed. En: endosperm-specific insertion, RS: radicle/shoot-specific insertion, R: radicle-specific insertion, Shoot: shoot-specific insertion, Leaf: leaf-specific insertion. (D) Percentage of leaf positions where the first *de novo mPing* insertion was found. Shoot: shoot-specific insertion, Leaf: leaf-specific insertion.

We performed TD analysis of *mPing* using genomic DNA samples extracted from endosperm, radicle, and leaf blades of eight progenies (S_1_) derived from a single parental EG4 plant (S_0_), and investigated the *mPing* transposition during ontogeny of rice plants (Figure 2A). One of the results of TD analysis using two selective bases is shown in Figure S3; all 16 possible primer combinations were analyzed. We examined *de novo* insertions in the same way as described above. Consequently, a total of 228 *de novo* insertions were detected. These insertions were divided into five groups (Figure 2B): (1) endosperm-specific insertions that appeared only in the endosperm sample, (2) radicle-specific insertions that appeared only in the radicle sample, (3) leaf-specific insertions that appeared only in one sample from the 1st to 3rd leaf blades, but not in the 4th and 5th leaf blades, (4) shoot-specific insertions that appeared in at least one sample of 1st, 2nd, and 3rd leaf blades, and in at least one sample of 4th and 5th leaf blades, and (5) radicle/shoot-specific insertions that appeared in both radicle and leaf blade samples. These *de novo* insertions were confirmed by sequence and locus-specific PCR analysis (Table S2 and Figure S4). Numbers of each insertion obtained in this study are summarized in Figure 2C. Plant development is divided roughly into three successive phases: embryogenesis, vegetative phase, and reproductive phase. If *mPing* transposed in the SAM of the S_0_ plant during vegetative and/or reproductive phases, the *de novo* insertions would segregate according to Mendel's law among the S_1_ progenies. We obtained no band that appeared in at least two S_1_ progenies and was not seen in the S_0_ plant. This indicates that the transmissible insertion of *mPing* to the next generation seldom (or never) arises during the vegetative and reproductive phases.

Flowering plants have evolved a unique reproductive process called double fertilization. In this process, either of two sperm cells in pollen fuses with either an egg cell or a central cell in the ovule, and then the egg cell fertilized with the sperm cell initiates embryogenesis [27]. In rice, the SAM and radicle are regionalized in the embryo 3 DAP, and three leaves and the radicle are already present in the mature embryo [28]. We detected only three radicle/shoot-specific insertions (Figure 2C), indicating that *mPing* scarcely transposes during the period from the onset of gametogenesis to the early stage (until 3 DAP) of embryogenesis. Among the 228 *de novo* insertions, 116 and 17 were shoot-specific and leaf-specific insertions, respectively (Figure 2C). This indicates that *mPing* actively transposes in the embryo during the period from the regionalization of SAM and radicle (at 3 DAP) to the formation of the 3rd leaf primordia (at 8 DAP). Of the 133 shoot- and leaf-specific insertions, 108 were of the 1st leaf blade (Figure 2D). Since the 1st leaf primordium is formed at 5 DAP, the most active phase of the *mPing* transposition was considered to be from 3 to 5 DAP. We detected a large number of radicle-specific insertions as well as shoot-specific insertions, and the sum of these insertions accounted for 90% of all insertions detected in this study (Figure 2C). Taken together, we concluded that *mPing* in EG4 most actively transposes in the 3 to 5 DAP embryo.

Endosperm is a triploid tissue that is produced by fusing a central cell containing two polar nuclei with one of two sperm cells in no particular order. The endosperm formation occurs in parallel with embryogenesis. The endosperm-specific insertions result from the *mPing* transposition occurred in either gametogenesis or endosperm formation. We observed only two endosperm-specific insertions (Figure 2C), supporting that *mPing* scarcely transposes during the period from the onset of gametogenesis to the early stage of embryogenesis. The relationship between the banding patterns obtained in TD analysis and the timing of *mPing* transposition is summarized in Figure S5.

### Inheritance of *de novo mPing* insertions to the next generation

In order for *mPing* to amplify, the *de novo* insertions must be transmitted to the next generation. We performed TD analysis using 12 progenies (S_2_) derived from the main culm and the primary tiller of a single selfed parent (S_1_) to investigate whether the *de novo* insertions detected in ontogenical analysis are inheritable (Figure S6). Both radicle-specific and leaf-specific insertions in the S_1_ plants were not detected in the S_2_ progenies (0 of 15, 0 of 2, respectively). In contrast, 85% (11 of 13) of the shoot-specific insertions that were detected in the S_1_ plants also appeared in the S_2_ progenies. This value (85%) is consistent with the estimated number of inheritable *de novo* insertions in our previous report [21]. Thus most of the *de novo* insertions that arose in the 3 to 5 DAP embryo were successfully inherited to the next generation.

### Excisions of *mPing* during ontogeny of EG4 plants

We have already determined the sites of all *mPing* insertions (1163 in total) in the EG4 genome [13], and have investigated *mPing* excisions in a small EG4 population using locus-specific primer pairs [29], [30]. Here we examined the timing of the *mPing* excision with locus-specific PCR using the genomic DNA samples that were used for the ontogenical analysis of the *de novo* insertion. We randomly chose 48 markers for this study (Table S3). We divided the *mPing* excisions into five types with the same criteria as those used for the *de novo* insertions: endosperm-, radicle-, leaf-, shoot-, and radicle/shoot-specific excisions (Figure S7). There were no endosperm-specific and radicle/shoot-specific excisions, indicating that no *mPing* transposition occurs during the period from the onset of gametogenesis to the early stage of embryogenesis. We detected seven radicle-specific, six leaf-specific, and three shoot-specific excisions. All shoot-specific excisions were detected from the 1st leaf blade sample. These results indicate that, like the *de novo* insertion, the *mPing* excision also occurs during the period from the regionalization of the SAM and radicle to the formation of the first leaf primordium, although we cannot completely rule out the possibility that these excisions occur also in somatic cells of mature tissues. Thus, in addition to the experimental results of the *de novo* insertion, we concluded that *mPing* of EG4 was most actively transposing in the 3 to 5 DAP embryo.

### Expression pattern of *Ping* in EG4

**Figure 3:**
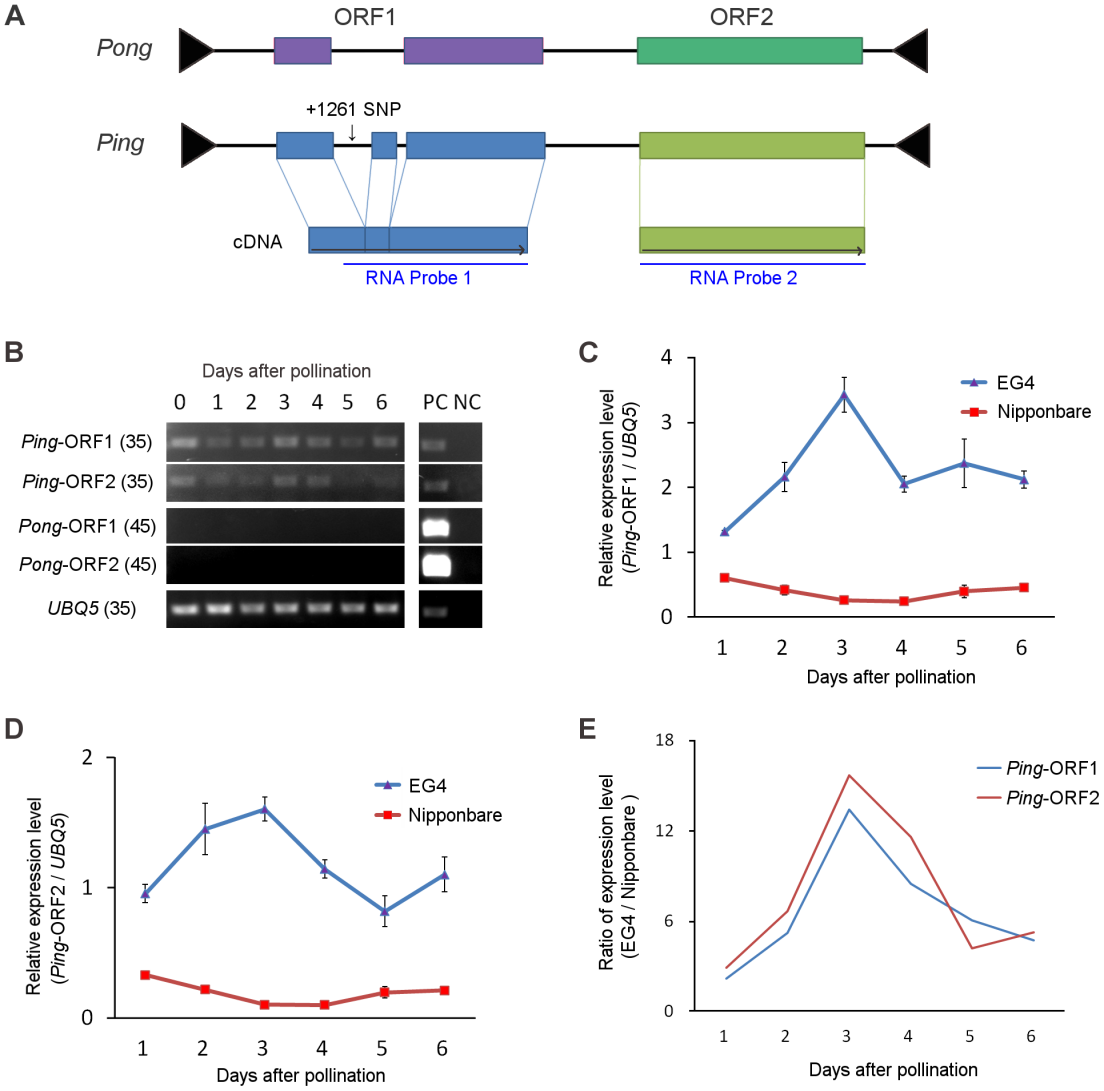
*Ping* expression during seed development. (A) Structure of the *Ping* and *Pong* elements. Terminal inverted repeats are indicated by black triangles. Boxes represent ORF1 and ORF2, respectively. Gray horizontal arrows indicate the direction of transcription. RNA probes used are indicated below the ORFs. (B) Reverse-transcription PCR analysis of *Ping*-ORF1, *Ping*-ORF2, *Pong*-ORF1, and *Pong*-ORF2. Numbers in parentheses are PCR cycle numbers. PC: positive control (0.1 ng genomic DNA), NC: negative control (non-reverse-transcribed RNA). (C) Real-time quantitative PCR of *Ping*-ORF1 and (D) *Ping*-ORF2. The expression level in the Nipponbare ovary just after pollination was set as 1. The results are presented as means of three biological replicates. Bars indicate SE. (E) The ratio of *Ping* expression level of EG4 to that of Nipponbare. The means in (C) and (D) were used for calculation.

Both *Ping* and *Pong* provide a Myb-like protein and a transposase, which are encoded by their ORF1 and ORF2, respectively (Figure 3A), and have been considered as autonomous elements responsible for the *mPing* transposition. We investigated the expression of *Ping*-ORF1, *Ping*-ORF2, *Pong*-ORF1, and *Pong*-ORF2 during embryogenesis to evaluate which autonomous element plays a predominant role in driving the *mPing* transposition in EG4. Reverse transcription-PCR analysis revealed that *Ping*-ORF1 and *Ping*-ORF2 constitutively expressed in the ovary during embryogenesis (Figure 3B). On the other hand, no transcriptions of *Pong*-ORF1 and *Pong*-ORF2 (Figure 3B) were observed. This strongly suggests that *Ping* predominantly controls the *mPing* transposition in EG4.

We performed real-time quantitative PCR (qPCR) analysis to compare the expression level of *Ping*-ORF1 and -ORF2 between EG4 and Nipponbare during embryogenesis. In all developmental stages from 1 to 6 DAP, the expression levels of both *Ping*-ORF1 and -ORF2 were higher in EG4 than in Nipponbare (Figure 3C, D). Since EG4 harbors seven copies of *Ping*, whereas Nipponbare has only one copy (Table S4), the difference in the expression levels between EG4 and Nipponbare is considered to be attributable to the different copy number of *Ping*. However, we found that *Ping* of EG4 showed different expression patterns from that of Nipponbare. In Nipponbare, the expression level of *Ping*-ORF1 and -ORF2 gradually declined until 3 DAP, and restored to the basal level at 6 DAP. In contrast, in EG4, the expression levels of both *Ping*-ORF1 and -ORF2 rapidly increased, with a peak at 3 DAP (Figure 3C, D). The ratio of relative expression level (EG4/Nipponbare) clearly demonstrated that *Ping* might be up-regulated in a developmental stage-specific manner in the ovary of EG4 (Figure 3E). Since *mPing* transposed during the period from 3 to 5 DAP, the rapid increase in *Ping* expression most likely drive the *mPing* transposition.

### Accumulation of *Ping* transcripts in the embryo triggers *mPing* transposition

**Figure 4:**
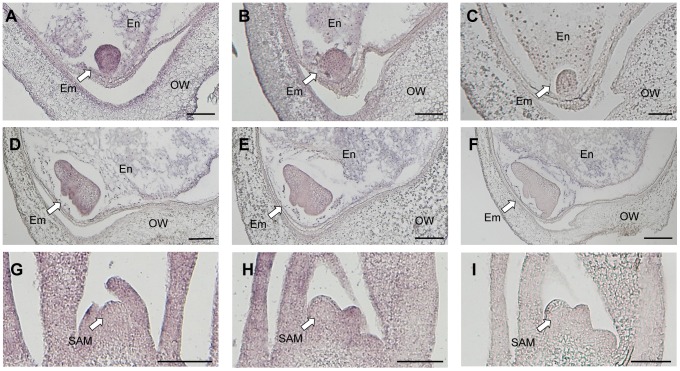
Detection of *Ping*-ORF1 spatial expression patterns by *in situ* hybridization analysis. Longitudinal sections through the ovary 3 days after pollination of (A, C) EG4 and (B) Nipponbare; the ovary 5 days after pollination of (D, F) EG4 and (E) Nipponbare; and the shoot apical meristem of (G, I) EG4 and (H) Nipponbare seedlings were hybridized with antisense (A, B, D, E, G, H) or sense (C, F, I) RNA probes. Little staining was obtained with the sense probe (F). Em: embryo, En: endosperm, OW: ovary wall, SAM: shoot apical meristem. Scale bars represent 100 µm.

We investigated the spatial expression pattern of *Ping* by *in situ* hybridization using *Ping*-specific probes. The probe positions were indicated in Figure 3A. The *Ping* transcripts were detected in all tissues, viz. embryo, endosperm, and ovary wall, in both EG4 and Nipponbare (Figure 4A–C, S8). Among the tissues, the 3 DAP embryo of EG4 yielded an exceptionally strong signal, indicating a high accumulation of *Ping* transcripts (Figure 4A), whereas the 5 DAP embryo showed a much lower accumulation of *Ping* transcripts in EG4 (Figure 4D–F). Such a drastic change in accumulation quantity of *Ping* transcripts with the advance of embryogenesis was consistent with the change in the expression quantity of *Ping* with the advance of embryogenesis, which was investigated by real-time qPCR (Figure 3C–E). These results suggest that the tissue- and developmental stage-specific accumulation of the *Ping* transcripts triggers *mPing* transposition at this stage in EG4. To confirm this hypothesis, we evaluated the spatial expression pattern of *Ping* in the SAM during the vegetative phase. As described above, *mPing* hardly transposes in the SAM during this phase. The *Ping* transcripts were detected in all tissues including the SAM, and, as expected, there was no obvious difference in the signal intensity between EG4 and Nipponbare (Figure 4G–I). Thus the *Ping* transcripts proved to accumulate developmental stage-specifically only in the tissue where *mPing* actively transposes. We therefore concluded that the high accumulation of *Ping* transcripts triggers the transposition of *mPing* in the 3 DAP embryo of EG4.

### SNP in an intronic region of *Ping*-ORF1

**Figure 5:**
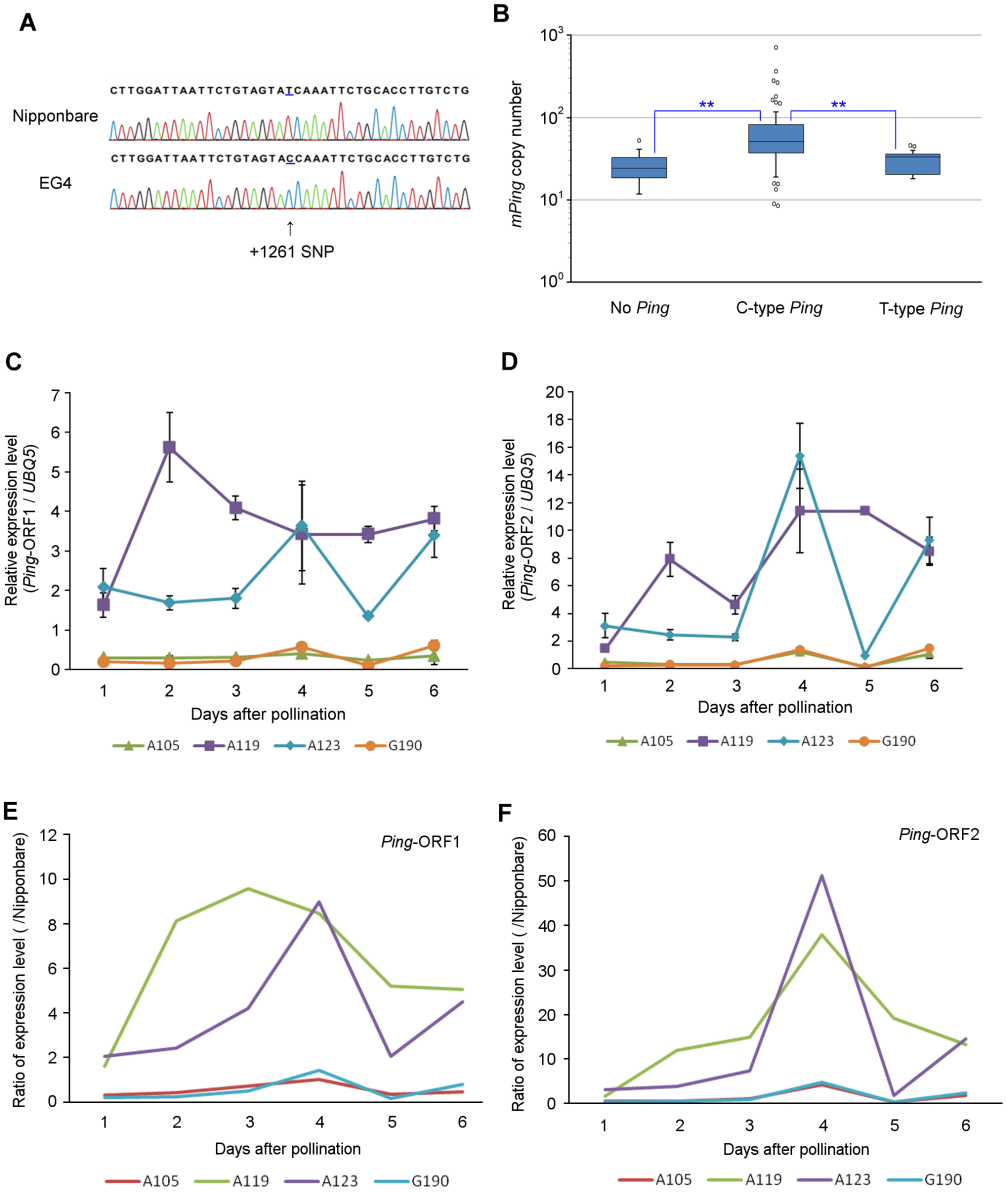
SNP in the first intronic region of *Ping*-ORF1. (A) Determination of the SNP sequence in the first intronic region of *Ping*-ORF1. The arrowhead indicates the position of the SNP. The number indicates the position of the *Ping* element. *Ping* harboring +1261C SNP and +1261T are named ‘C-type’ and ‘T-type’ *Ping*, respectively. (B) Box plots of *mPing* copy number in AG lines. The top and bottom of the boxes mark the first and third quartiles, respectively. The center line represents the median, and the whiskers show the range of observed values within 1.5 times the interquartile range from the hinges. Values beyond 1.5 times the interquartile range from the nearest hinge are marked by open circles. ‘No *Ping*’, ‘C-type *Ping*,’ and ‘T-type *Ping*’ indicate the groups having no *Ping*, C-type *Ping*, and T-type *Ping*, respectively. Expression of (C) *Ping*-ORF1 and (D) *Ping*-ORF2 during embryogenesis in *mPing*-active strains (A119 and A123) and *mPing*-inactive strains (A105 and G190). The results are presented as means of three biological replicates. Bars indicate SE. The ratio of (E) *Ping*-ORF1 and (F) -ORF2 expression level of A105, A119, A123, and G190 to that of Nipponbare. The means in (Fig. 3C) and (Fig. 3D) were used for calculation.

EG4 has seven *Ping* elements (*Ping-1* to *-7*), whereas Nipponbare has only one (*Ping-N*) (Table S4). When we sequenced and compared all *Ping* elements, a single nucleotide polymorphism (SNP) in the first intronic region of *Ping*-ORF1 was detected between EG4 and Nipponbare (Figure 5A). *Ping-N* has a ‘T’ nucleotide on the SNP region, whereas all *Ping* elements in EG4 have a ‘C’ nucleotide. We named the former ‘T-type *Ping*’ and the latter ‘C-type *Ping*’.

In addition to EG4, several Aikoku and Gimbozu landraces (hereafter AG strains) are known to exhibit high *mPing* activity [21]. We investigated the SNP-type of *Ping* and the copy number of *Ping* and *mPing* in 93 AG strains, and evaluated the effect of C-type *Ping* on the *mPing* activity. These 93 AG strains were divided into three groups according to the SNP-type of the *Ping* allele (Table S4): strains harboring C-type *Ping*; strains harboring T-type *Ping*; and strains harboring no *Ping*. The strains with C-type *Ping* had more *mPing* copies than those with T-type *Ping* or no *Ping* (Figure 5B, Steel–Dwass test, p<0.01). This implies that the C-type *Ping* could drive the *mPing* transposition. We further investigated the expression patterns of *Ping*-ORF1 and -ORF2 in two *mPing*-active strains (A119 and A123) and two *mPing*-inactive strains (A105 and G190) during embryogenesis (from 1 to 6 DAP). A119 and A123 have six and ten copies of C-type *Ping*, respectively, and both A105 and G190 have one copy of T-type *Ping* (Table S4). Expression analysis revealed that A105 and G190 kept low expression levels of *Ping*-ORF1 and -ORF2, whereas A119 and A123 showed high expression levels with a peak around 3 DAP (Figure 5C–F). This indicates that, in EG4, A119, and A123, the developmental stage-specific expression of *Ping* is controlled by the same factor(s) described in the Discussion.

## Discussion

Our final goal was to elucidate how MITEs attain their high copy numbers in the genome. To this end, we chose *mPing*, which is the only active MITE identified in rice, as a material and analyzed the timing of *mPing* transposition in the *mPing*-active strain EG4. Consequently, we successfully found one mechanism of the *mPing* amplification; *mPing* most actively transposes during the period from the regionalization of the SAM and radicle to the formation of the first leaf primordium (3 to 5 DAP) by the developmental stage-specific up-regulation of the autonomous element *Ping*.

The transpositions of TEs are categorized into germinal and somatic types according to the type of cells where the transposition takes place. *LORE1a* in *Lotus japonicus* is activated in plants regenerated from de-differentiated cells and transposes in male germ cells by the pollen grain-specific *LORE1a* transcription, resulting in the asymmetric transposition of *LORE1a* in the reciprocal crosses between the active and non-active lines [31]. *Tag1* in Arabidopsis shows germinal transposition activity in both male and female germ cells. Consequently, the reciprocal crosses show symmetric transposition of *Tag1* [32]. These results demonstrate that the transposition activity in reciprocal crosses reflects the tissue specificity of germinal transposition. In this study, reciprocal crosses between EG4 and Nipponbare showed the same *mPing* transposition activity, which may suggest that *mPing* in EG4 transposes in both male and female germ cells. However, we obtained only a few *de novo* endosperm-specific and radicle/shoot-specific insertions in the ontogenical analysis, although we detected a number of *de novo* shoot-specific and radicle-specific insertions. We therefore concluded that most *mPing* transposes not in germ cells but in somatic cells after pollination. Somatic transposition that occurs at the late stage of plant development often produces spotted and striped segments in tissues, such as maize seed coat variegation caused by *Mutator* excision from the *bz2* gene [33], [34] and rice leaf color variegation by *nDart1*excision from the *OsClpP5* gene [35]. In animals, somatic transposition is seldom transmitted to the next generation because germ cells are set aside from somatic cells at the early stage of embryogenesis. On the other hand, in plants, germ cells are generated from somatic cells at the reproductive stage. In rice, gametes are generated in the SAM; therefore, somatic transposition that occurred in the SAM can be transmitted to the next generation via gametes. In this study, we revealed that most *mPing* elements transposed in somatic cells of the embryo during the period from 3 to 5 DAP. Being a class II TE that transposes by a cut-and-paste mechanism, *mPing* is expected to be eliminated from genomic DNA with a certain frequency. However, a previous report demonstrated that the *mPing* excision sites would be repaired by utilizing a copy of *mPing* from either the sister chromatid or from the homologous chromosome [29]. The *mPing* excision site cannot be repaired if *mPing* transposes in germ cells, which are haploid. It is therefore considered that the somatic transposition of *mPing* is an important factor for *mPing* amplification in the genome.

The autonomous elements *Ping* and *Pong* mediate *mPing* transposition in the rice genome. Many *japonica* cultivars, including EG4 and Nipponbare, have both *Ping* and *Pong*. This study demonstrated that *Ping* plays a predominant role in *mPing* transposition in EG4. However, a heterologous expression assay using Arabidopsis and yeast showed that *Pong* had a higher catalytic capacity for *mPing* transposition than *Ping* [22], [23]. Furthermore, *mPing* transposition was observed under stress conditions in several rice cultivars harboring only *Pong* [14], [17], [19]. In this study, however, we detected very low expression of *Pong* through the development of rice plants, indicating that *Pong* would be epigenetically silenced at the transcriptional level in EG4. In contrast, *Ping* constitutively expressed in all organs including the SAM and embryo. Nevertheless, *mPing* could be transposing most actively in the embryo during the period from 3 to 5 DAP. Since the stage-specific up-regulation of *Ping* was observed during the period of *mPing* transposition, we hypothesized that the expression level of *Ping* needed to exceed a certain threshold of *mPing* transposition.

All *mPing*-active strains (EG4, A119, and A123) showed higher expression of *Ping* with a peak around 3 DAP than the *mPing*-inactive strains (Nipponbare, A105, and G190). Although further experiments are needed to elucidate the mechanism of developmental stage-specific up-regulation of *Ping* expression, we propose two hypotheses: (1) position- and dosage-effect, and (2) effect of SNP. The details of the hypotheses are as follows.

### Position- and dosage-effect

Chromosomal position and copy number of TE often affect the transposition activity. The former is known as ‘position effect’ and the latter as ‘dosage effect’. Eight independent *Tam3* copies residing in the *Antirrhinum majus* genome show different transposition activities from each other [36]. In Arabidopsis, germinal reversion frequency of *Tag1* increases in proportion to its copy number [32]. The *mPing*-inactive strains Nipponbare, A105, and G190 have only one *Ping* at the same locus, whereas the *mPing*-active strains EG4, A119, and A123 have respectively seven, six, and ten copies of *Ping* at different loci except for the *Ping-1* locus. Furthermore, the expression pattern of *Ping* showed slight variation among the *mPing*-active strains harboring only C-type *Ping*. These results suggest that the developmental stage-specific up-regulation of *Ping* expression is probably regulated by the position-effect and/or the dosage-effect.

### Effect of SNP

Intronic SNPs are known to cause drastic effects on gene expression. In humans, an intronic SNP in *SLC22A4* affects transcriptional efficiency *in vitro*, owing to an allelic difference in affinity to the transcriptional factor RUNX1 [37]. Furthermore, a SNP located in the intronic enhancer region of the thyroid hormone receptor β gene enhances pituitary cell-specific transcriptional activity [38]. In this study, we demonstrated that a SNP is present in the intronic region of *Ping*-ORF1, and *Ping* elements in the AG strains were categorized into either T-type or C-type *Ping* according to the SNP-type. Since all strains that showed a peak in the expression analysis had only C-type *Ping*, the intronic SNP might influence the developmental stage-specific up-regulation of *Ping* expression. T-type *Ping* was present in 14 AG strains as one copy, and its chromosomal location did not differ among strains. In contrast, the copy number of C-type *Ping* varied from one to ten, and their chromosomal locations, except for *Ping*-1, differed from each other. These results indicate that T-type *Ping* has lost its activity, whereas C-type *Ping* may be still active in the rice genome. Furthermore, we found that the copy number of *mPing* was significantly larger in strains harboring C-type *Ping* than in strains harboring T-type *Ping*. This strongly supports that C-type SNPs in the intronic region of *Ping* contribute to the amplification of *mPing*, presumably by the developmental stage-specific up-regulation of *Ping* expression.

Since the transposition of TEs often damages the host genome, TEs with high transposition activity are targeted by the silencing mechanisms. Nevertheless, MITEs amplify to very high copy numbers not only in plant genomes but also in animal genomes. Very little is known about how MITEs attain their high copy numbers by escaping the silencing mechanism. The transposition of *mPing* is transiently induced by various stresses [14]–[18], indicating that the activity of *mPing* is suppressed by the silencing mechanisms in many cultivars. Thus, *mPing* must overcome the silencing mechanism in order to maintain the transposition activity under natural growth conditions. Our results revealed that *mPing* in EG4 was mobilized by the sufficient supply of *Ping* transcripts produced only during the period of *mPing* transposition. This stage-specific activation is thought to be a strategy of the *mPing* family to amplify *mPing* by escaping from the silencing mechanism of the host genome. Since no active MITEs other than *mPing* so far have been identified, it is very difficult to elucidate if the other MITEs also attain their high copy numbers in the same way as *mPing* amplifies. Given that the other active MITEs are identified, however, our study will help to understand their amplification mechanisms. Our previous study documented the generation of new regulatory networks by a subset of *mPing* insertions that render adjacent genes stress inducible [13]. In addition to *mPing*, other MITEs also contribute to gene and genome evolution via providing new promoter regulatory sequences, transcriptional termination elements, and new alternative exons [39], suggesting that the amplification of MITEs causes gene and genome evolution. Our results provide clues to further understand not only the amplification mechanism of MITEs but also the co-evolution of MITEs and the host genome.

## Materials and Methods

### Plant materials and sampling

EG4 (cultivar Gimbozu), Nipponbare, and 94 Aikoku/Gimbozu landraces were used in this study (Table S4). Aikoku/Gimbozu landraces were provided from the GenBank project of the National Institute of Agrobiological Science, Ibaraki, Japan. Reciprocal crosses between EG4 and Nipponbare were made in a green house. Before pollination, all anthers were removed from the flowers of maternal plants. The pollinated flowers were covered with protective bags to prevent outcrossing until harvest. After harvesting, success of crosses was checked with the molecular markers. For ontogenical analysis, eight progenies of EG4 (S_1_) derived from a single parental plant (S_0_) were grown in a greenhouse, and all S_2_ seeds were harvested. For S_1_ plants, each seed was cut into two halves, and the half including the embryo was germinated and the other was sampled. After germination, the radicle and the 1st, 2nd, 3rd, 4th, and 5th leaf blades were sampled. The second leaf was collected from S_0_ and S_2_ plants. For estimation of *Ping* and *mPing* copy numbers, eight bulked plants were sampled. For RNA extraction, ovaries before pollination and ovaries at 1, 2, 3, 4, 5, and 6 DAP were collected. All samples were immediately frozen in liquid nitrogen and stored at −80°C until use.

### DNA extraction and transposon display

DNA extraction and transposon display was performed following a published protocol [30]. For DNA extraction from endosperm, we used GM quicker 2 (Nippon Gene).

### Locus-specific PCR

Sequencing of *mPing*-flanking fragments excised from transposon display gels and primer design were performed following a published protocol [30]. The genomic locations of the *mPing* insertion sites were forecasted by a BLAST search in the Rice Annotation Project Database (RAP-DB; http://rapdb.dna.affrc.go.jp/) [40], [41] using *mPing* flanking sequences as queries. To prepare enough templates for PCR, whole genome amplification was performed using an illustra GenomiPhi V2 Kit (GE Healthcare). *mPing* excision was detected by PCR with *mPing*-sequence characterized amplified region (SCAR) markers [29]. PCR was performed in 10-µl reaction volumes containing 10 ng of the template DNA, 5 µl of GoTaq Green Master mix (Promega), 5% DMSO, and 0.25 µM of each primer (Table S3). PCR conditions were as follows: 94°C for 3 min; 40 cycles of 98°C for 10 s, 57°C for 30 s, and 72°C for 45 s; and 72°C for 5 min. To detect the presence of *Ping-N*, *-1*, *-2*, *-3*, *-4*, *-5*, *-6*, and *-7*, eight *Ping*-SCAR markers were used. The genomic locations of the *Ping* insertion sites were referred from a previous report [42]. For detection of the *Ping-1* allele, PCR was performed in 10-µl reaction volumes containing 10 ng of template DNA, 0.2 U of KOD FX Neo (Toyobo), 1×PCR buffer for KOD FX Neo (Toyobo), and 0.2 µM of each primer (Table S5). PCR conditions were as follows: 94°C for 3 min; 35 cycles of 98°C for 10 s, 60°C for 30 s, and 68°C for 90 s; and 72°C for 1 min. For detection of *Ping-N*, *-2*, *-3*, *-4*, *-5*, *-6*, and *-7* alleles, PCR was performed in 10-µl reaction volumes containing 10 ng of template DNA, 5 µl of GoTaq Green Master mix (Promega), 5% DMSO, and 0.25 µM of each primer (Table S5). PCR conditions were as follows: 94°C for 3 min; 35 cycles of 98°C for 10 s, 60°C for 30 s, and 72°C for 45 s; and 72°C for 1 min.

### RNA isolation and expression analysis

Total RNA was isolated using TriPure isolation reagent (Roche) and digested using RNase-free DNase (TaKaRa). First strand cDNA was synthesized using a Transcriptor first strand cDNA synthesis kit (Roche). For reverse transcription PCR, PCR was performed in 10 µl reaction volumes containing cDNA generated from 4 ng total RNA, 0.2 U of KOD FX Neo (Toyobo), 1×PCR buffer for KOD FX Neo (Toyobo), and 0.5 µM of each primer. PCR conditions were as follows: 94°C for 3 min; 35 or 45 cycles of 98°C for 10 s, 60°C for 10 s, and 68°C for 10 s. Relative quantification of *Ping*-ORF1 and *Ping*-ORF2 were calculated by the 2^−ΔΔCT^ method [43] using Light cycler 1.5 (Roche). The *UBQ5* gene was used as the calibrator gene. The thermal profile consisted of 10 min at 95°C; and 45 cycles of 4 s at 95°C, 10 s at 60°C, and 1 s at 72°C. Amplification data were collected at the end of each extension step. The primer pairs used in this study are listed in Table S6.

### Paraffin sectioning and *in situ* hybridization

Plant samples were fixed with 4% (w/v) paraformaldehyde and 1% Triton X in 0.1M sodium phosphate buffer for 48 h at 4°C. They were then dehydrated in a graded ethanol series, substituted with 1-butanol, and embedded in Paraplast Plus. The samples were sectioned at 8-µm thickness using a rotary microtome. Fragments of *Ping*-ORF1 (1091 bp) and *Ping*-ORF2 (1368 bp) were cloned into pBlueScript SK+ (Stratagene) and sequenced. For digoxigenin-labeled antisense/sense RNA probe synthesis, *in vitro* transcription was performed using T7 RNA polymerase and T3 RNA polymerase. *In situ* hybridization and immunological detection with alkaline phosphatase were performed according to Kouchi and Hata [44].

### SNP detection

PCR was performed in 10-µl reaction volumes containing 10 ng of template of DNA, 5 µl of GoTaq Green Master mix (Promega), 5% DMSO, and 0.25 µM of each primer. PCR conditions were as follows: 94°C for 3 min; 35 cycles of 98°C for 10 s, 60°C for 30 s, and 72°C for 30 s; and 72°C for 1 min. PCR primers used in this study are listed in Table S6. Because the original sequence contained an *Afa* I restriction site, one mutation was introduced into the reverse primer. The 5-µl PCR products were mixed with 5 µl restriction mixture containing 1 U *Afa* I (TaKaRa), 33 mM Tris-acetate (pH 7.9), 10 mM Mg-acetate, 0.5 mM Dithiothreitol, 66 mM K-acetate, and 0.01% (w/v) bovine serum albumin. After 16 h incubation at 37°C, DNA gel electrophoresis was performed. PCR products (502 bp) including +1261T SNP were not digested, whereas PCR products including +1261C SNP were digested into two fragments (352 bp and 150 bp).

### Estimation of *Ping* and *mPing* copy number

To determine the copy number of *Ping* by Southern blot analysis, genomic DNA samples were digested with *Eco* RI restriction enzyme. These samples were loaded onto an agarose gel, separated by electrophoresis, blotted onto a nylon membrane, and probed with the *Ping* fragment. The *mPing* copy number was determined by real-time quantitative PCR as described previously [45] with little modification. Quantitative PCR was performed using the LightCycler 480 system (Roche). PCR was performed in 20 µl reaction volumes containing 5 µl genomic DNA (0.4 ng/µl), 1×LightCycler 480 SYBR Green I Master mix (Roche), and 0.5 µM of each primer. Specificity of the amplified PCR product was assessed by performing a melting curve analysis on the LightCycler 480 system.

## Supporting Information

Figure S1 Verification of the reciprocal crosses between EG4 and Nipponbare. (A) Locus-specific PCR analysis of *Ping* with locus-specific markers. The maker names and cross combinations are indicated at the top of the profiles. (B) Locus-specific PCR analysis of *mPing* in Nipponbare genome. The genomic location of the *mPing* insertions and cross combinations are indicated at the top of the profiles. G and F indicate parental EG4 plants and the F_1_ plants, respectively. Lane M: DNA size marker (Gene Ladder 100, Nippon Gene), Lane N: Nipponbare. Black and white arrowheads show the bands indicating the presence and absence of *Ping*/*mPing*, respectively.
(TIF)

Figure S2 Locus-specific PCR analysis of *de novo mPing* insertions in F_1_ progenies. Ten representative results are shown. The genomic locations of the *mPing* insertions and cross combinations are indicated at the top of the profiles. G and F indicate parental EG4 and the F_1_ plants, respectively. Lane M: DNA size marker (Gene Ladder 100, Nippon Gene), Lane N: Nipponbare. Black and white arrowheads show the bands indicating the presence and absence of *mPing*, respectively.
(TIF)

Figure S3 Ontogenical analysis of *mPing* transposition in EG4 by transposon display. Eight progenies (S_1_) were derived from a single parental EG4 plant (S_0_). The 2nd leaf blade of the S_0_ plant and the endosperm, radicle, and leaf blades of each S_1_ plant were sampled and subjected to transposon display. White, red, and green arrowheads indicate shoot-, radicle-, and leaf-specific insertions, respectively. The rice plant has alternate distichous leaves; therefore, we analyzed the insertion in both [n+1]th and [n+2]th leaves to confirm whether the insertion detected in [n]th leaf is leaf-specific or shoot-specific. But we did not investigate the specificity of the insertions detected in the 4th and 5th leaves using their upper leaves. For this reason, we did not categorize such insertions and marked with the gray arrowhead. E: endosperm; R: radicle; L1–L5: 1st to 5th leaf. For progeny 2–8, samples are applied in the same order as for progeny 1.
(TIF)

Figure S4 Locus-specific PCR analysis of *de novo mPing* insertions in various tissues of a single EG4 plant. Eight representative results are shown. The genomic locations of the *mPing* insertions and insertion types are indicated at the top of the profiles. Lane M: DNA size marker (Gene Ladder 100, Nippon Gene), Lane N: Nipponbare, Lane E: endosperm, Lane R: radicle, Lane L1–L5: 1st to 5th leaf. Black and white arrowheads show the bands indicating the presence and absence of *mPing*, respectively.
(TIF)

Figure S5 Schematic representation of the relationship between banding patterns obtained in transposon display and the timing of *mPing* transposition. If *mPing* transposes in the period indicated by the red bar, the schematic banding patterns indicated by the arrows will be obtained. E: endosperm, R: radicle, L1–L5: 1st to 5th leaf blade, DAP: days after pollination, SAM: shoot apical meristem.
(TIF)

Figure S6 Inheritance of *de novo mPing* insertions in EG4. S_2_ plants derived from the main culm and the primary tiller of a single S_1_ plant were assayed. The shoot-specific insertion in the S_1_ plant (white arrowhead) was inherited by S_2_ plants, whereas the radicle-specific insertion (red arrowhead) was not. E: endosperm; R: radicle; L1–L5: 1st to 5th leaf.
(TIF)

Figure S7 *mPing* excisions in EG4. *mPing* excisions were detected by locus-specific PCR using the genomic DNA samples that were used for the ontogenical analysis of the *de novo* insertion. We analyzed 48 loci. Black and white arrowheads show the bands indicating the presence and absence of *mPing*, respectively. Figures indicate (A, B) shoot-specific excisions, (C, D) leaf-specific excisions, and (E) radicle-specific excision. G: EG4 (S_0_ plant); N: Nipponbare; E: endosperm; R: radicle; L1–L5: 1st to 5th leaf.
(TIF)

Figure S8 Detection of *Ping*-ORF2 spatial expression patterns by *in situ* hybridization analysis. Longitudinal sections through the ovary 3 days after pollination of (A) EG4 and (B, C) Nipponbare were hybridized with (A, B) antisense or (C) sense RNA probes.
(TIF)

Table S1 *De novo mPing* insertion sites detected in F_1_ progenies.
(XLSX)

Table S2 *De novo mPing* insertion sites detected in various tissues of a single EG4 plant.
(XLSX)

Table S3 *mPing*-SCAR markers used in this study.
(XLSX)

Table S4 Plant materials used in this study.
(XLSX)

Table S5 *Ping*-SCAR markers used in this study.
(XLSX)

Table S6 Primer pairs used in this study.
(XLSX)
