# Supplementary figures and images for: Early Embryogenesis-Specific Expression of the Rice Transposon Ping Enhances Amplification of the MITE mPing
Source: PLoS Genet. 2014 Jun 12;10(6):e1004396. doi: 10.1371/journal.pgen.1004396 (PMC4055405; doi:10.1371/journal.pgen.1004396)

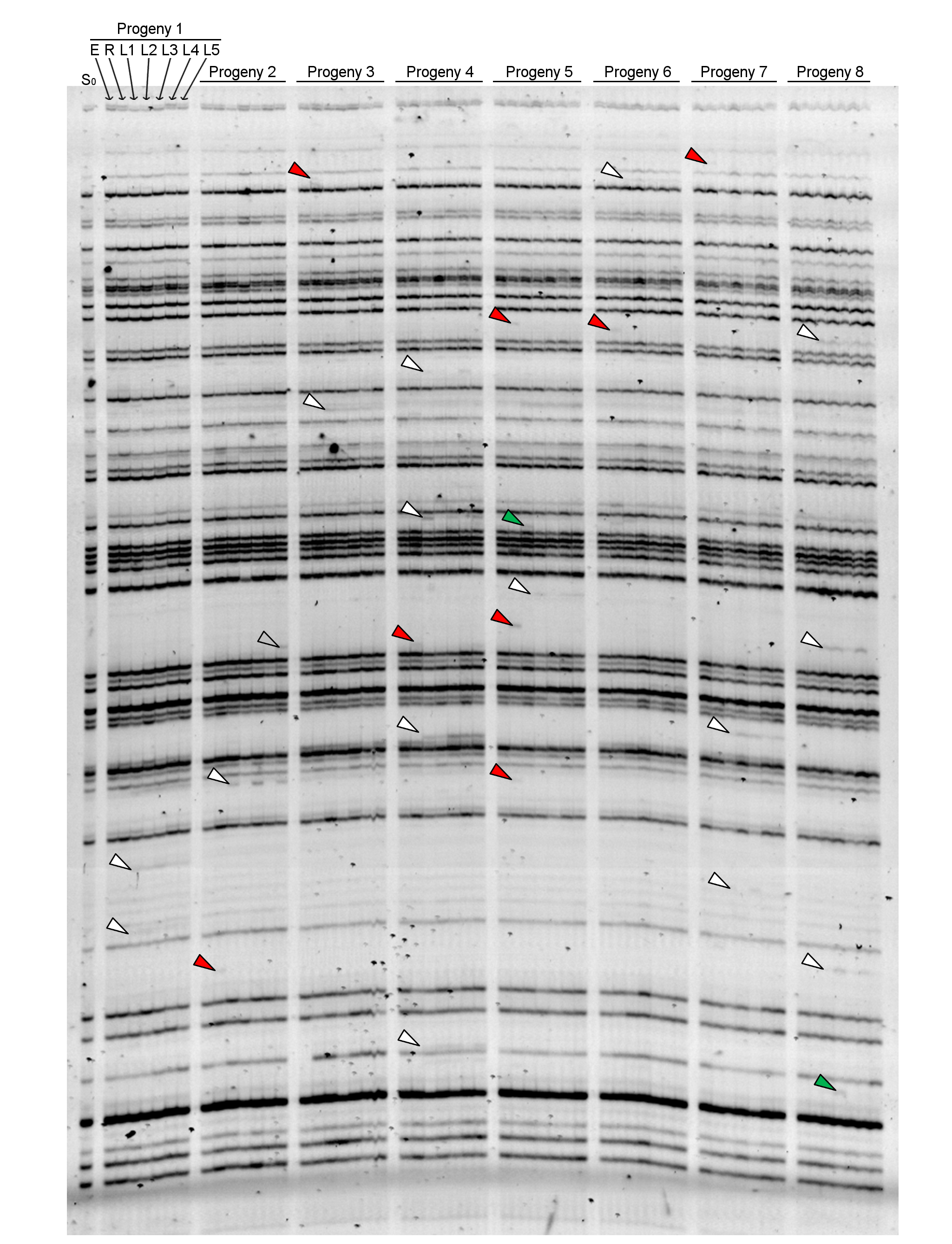

Supplement: Figure S3 — Ontogenical analysis of mPing transposition in EG4 by transposon display. Eight progenies (S1) were derived from a single parental EG4 plant (S0). The 2nd leaf blade of the S0 plant and the endosperm, radicle, and leaf blades of each S1 plant were sampled and subjected to transposon display. White, red, and green arrowheads indicate shoot-, radicle-, and leaf-specific insertions, respectively. The rice plant has alternate distichous leaves; therefore, we analyzed the insertion in both [n+1]th and [n+2]th leaves to confirm whether the insertion detected in [n]th leaf is leaf-specific or shoot-specific. But we did not investigate the specificity of the insertions detected in the 4th and 5th leaves using their upper leaves. For this reason, we did not categorize such insertions and marked with the gray arrowhead. E: endosperm; R: radicle; L1–L5: 1st to 5th leaf. For progeny 2–8, samples are applied in the same order as for progeny 1. (TIF) [file pgen.1004396.s003.tif]

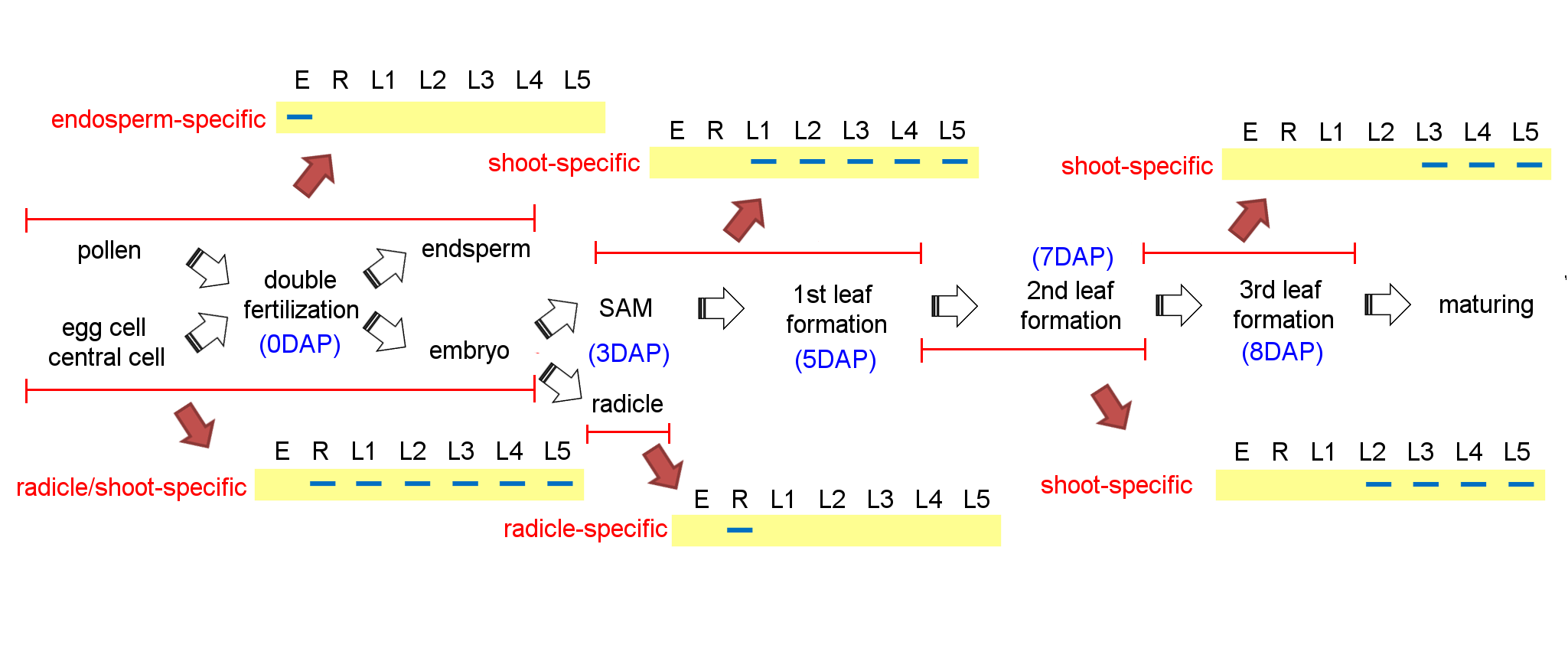

Supplement: Figure S5 — Schematic representation of the relationship between banding patterns obtained in transposon display and the timing of mPing transposition. If mPing transposes in the period indicated by the red bar, the schematic banding patterns indicated by the arrows will be obtained. E: endosperm, R: radicle, L1–L5: 1st to 5th leaf blade, DAP: days after pollination, SAM: shoot apical meristem. (TIF) [file pgen.1004396.s005.tif]

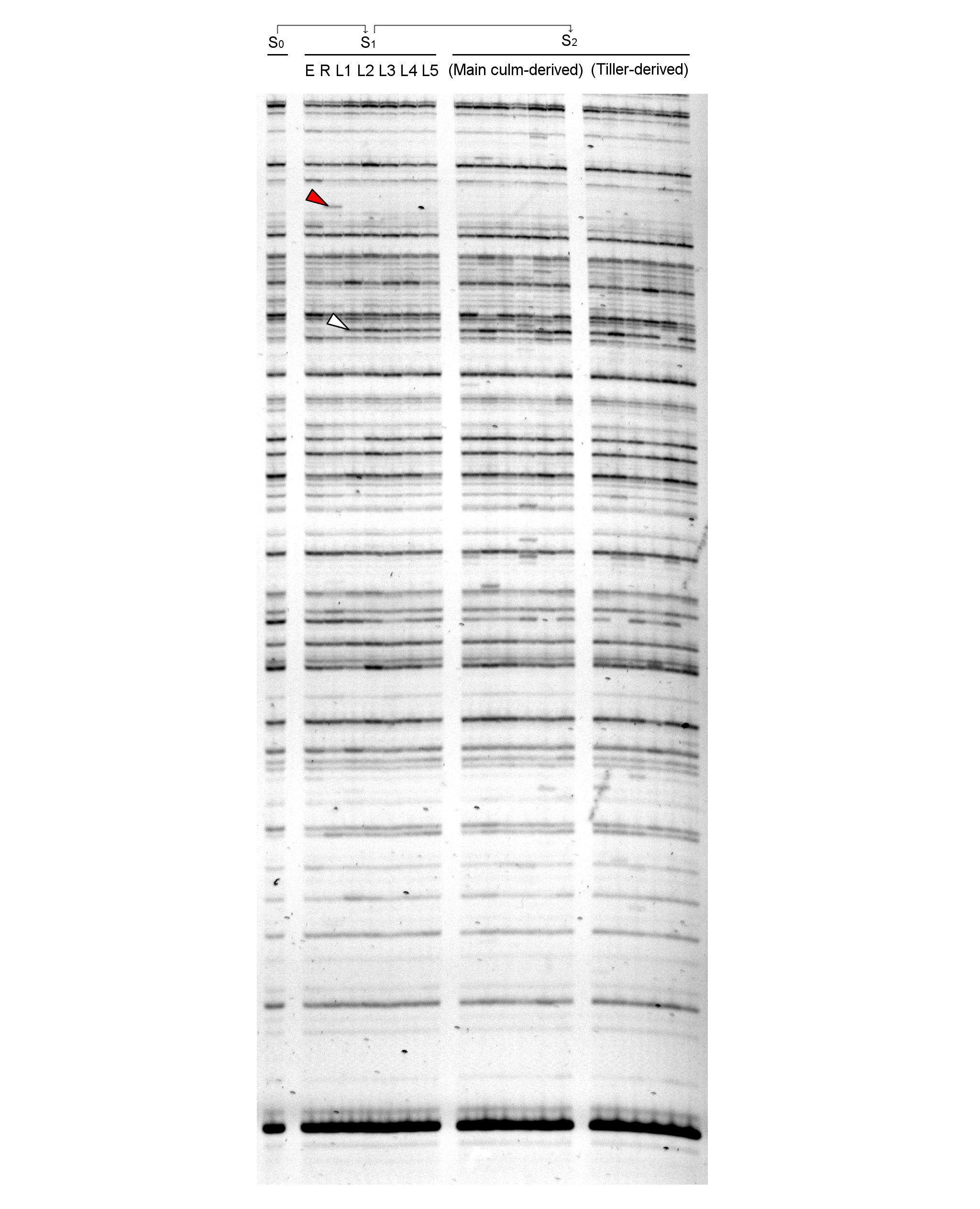

Supplement: Figure S6 — Inheritance of de novo mPing insertions in EG4. S2 plants derived from the main culm and the primary tiller of a single S1 plant were assayed. The shoot-specific insertion in the S1 plant (white arrowhead) was inherited by S2 plants, whereas the radicle-specific insertion (red arrowhead) was not. E: endosperm; R: radicle; L1–L5: 1st to 5th leaf. (TIF) [file pgen.1004396.s006.tif]

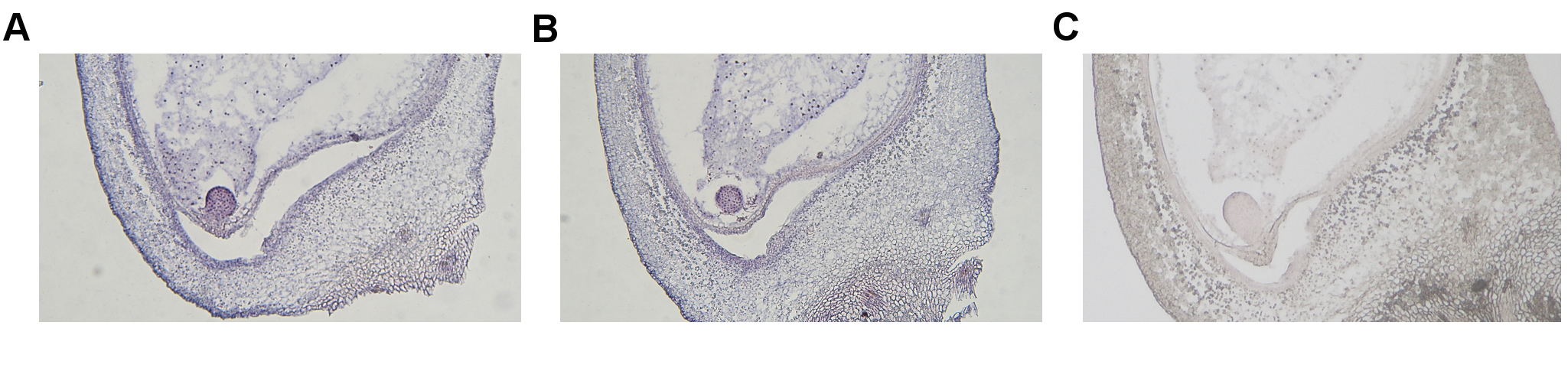

Supplement: Figure S8 — Detection of Ping-ORF2 spatial expression patterns by in situ hybridization analysis. Longitudinal sections through the ovary 3 days after pollination of (A) EG4 and (B, C) Nipponbare were hybridized with (A, B) antisense or (C) sense RNA probes. (TIF) [file pgen.1004396.s008.tif]
